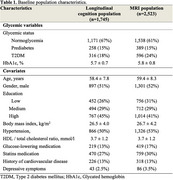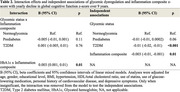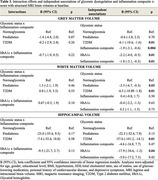# The association of glycemic dysregulation with cognitive decline and structural MRI brain volumes in The Maastricht Study – A role for peripheral inflammation?

**DOI:** 10.1002/alz70857_100484

**Published:** 2025-12-24

**Authors:** Veerle van Gils, Carlos Gómez‐Martínez, Miranda T. Schram, Gabriëlla A.M. Blokland, Giuseppe Fanelli, Janita Bralten, Nina Roth Mota, Søren Dalsgaard, Jacobus F.A. Jansen, Walter H. Backes, David E.J. Linden, Anke Wesselius, Tos T.J.M. Berendschot, Kay Deckers, Carla J.H. van der Kallen, Annemarie Koster, Marleen M.J. van Greevenbroek, Sebastian Köhler, Jordi Salas Salvadó, Pieter Jelle Visser, Stephanie J. B. Vos, Willemijn J. Jansen, PRIME consortium

**Affiliations:** ^1^ Alzheimer Center Limburg, School for Mental Health and Neuroscience, Maastricht University, Maastricht, Limburg, Netherlands; ^2^ Universitat Rovira i Virgili, Departament de Bioquímica i Biotecnologia, ANUT‐DSM group, Reus, Spain; ^3^ Unitat de Nutrició Humana, Institute of Health Pere Virgili (IISPV), Reus, Spain; ^4^ CIBER Fisiopatología Obesidad y Nutrición (CIBERobn), Instituto de Salud Carlos III, Reus, Spain; ^5^ Mental Health and Neuroscience Research Institute, Maastricht University, Maastricht, Netherlands; ^6^ Department of Internal Medicine, Cardiovascular Research Insitute CARIM, Maastricht University, Maastricht, Netherlands; ^7^ Department of Human Genetics, Radboud University Medical Center, Donders Institute for Brain, Cognition and Behaviour, Nijmegen, Netherlands; ^8^ National Centre for Register‐based Research, Department of Economics and Business, School of Business and Social Sciences, Aarhus University, Aarhus, Denmark; ^9^ Child and Adolescent Mental Health Center, Copenhagen University Hospital, Mental Health Services CPH, Copenhagen, Denmark; ^10^ Dept of Clinical Medicine, University of Copenhagen, Copenhagen, Denmark; ^11^ Department of Epidemiology, NUTRIM Institute of Nutrition and Translational Research in Metabolism, Maastricht University, Maastricht, Netherlands; ^12^ Alzheimer Center Limburg, Mental Health and Neuroscience Research Institute, Maastricht University, Maastricht, Netherlands; ^13^ Department of Social Medicine, CAPRHI Care and Public Health Research Institute, Maastricht, Netherlands; ^14^ Alzheimer Center and Department of Neurology, Amsterdam Neuroscience Campus, VU University Medical Center, Amsterdam, Netherlands

## Abstract

**Background:**

It remains unclear whether the combination of glycemic dysregulation and peripheral inflammation is synergistically associated with accelerated cognitive decline and decreased brain volumes.

**Method:**

In 2,832 persons from The Maastricht Study (*N* = 1,745 with longitudinal cognition measures, *N* = 2,523 with MRI), we assessed the presence of synergistic effects of glycemic status (normoglycemia, prediabetes, type 2 diabetes mellitus, T2DM) and glycated hemoglobin levels (HbA1c, %) with an inflammation composite z‐score measured in plasma (CRP, IL‐6, IL‐8, SAA, TNF‐a, SICAM‐1) on (1) z‐scores of global cognitive function over 9 years (mean of processing speed, executive function, and memory) using linear mixed modelling, as well as (2) cross‐sectional MRI measures of global grey matter, global white matter, and hippocampal volumes (ml), using linear regression. Analyses were adjusted for various covariates (Table 1).

**Result:**

Demographics are shown in Table 1. Longitudinal cognition analyses showed no significant interaction between glycemic status and the inflammation composite z‐score with cognitive decline (Table 2). Independent associations of T2DM (*p* <0.001) and the inflammation composite z‐score (*p* = 0.01), but not prediabetes, with increased global cognitive decline over 9 years were observed. Additionally, we found an interaction of HbA1c and the inflammation composite z‐score on global cognitive decline (*p* = 0.01). In persons with lower inflammation composite z‐scores, higher HbA1c was associated with greater cognitive decline, as compared to those with higher inflammation.

In cross‐sectional MRI analyses no interactions were found (Table 3). Independent associations of T2DM (*p* = 0.01), HbA1c (*p* = 0.01), and inflammation composite z‐score (*p* <0.01 in both models), but not prediabetes, with grey matter volume were observed. T2DM (*p* = 0.01) and prediabetes (*p* = 0.03), but not inflammation composite z‐score, were associated with lower white matter volume. A similar pattern was found for hippocampal volume, where T2DM (*p* = 0.01) and HbA1c (*p* = 0.04), but not prediabetes or inflammation composite z‐score, were associated with lower hippocampal volume.

**Conclusion:**

Together, our findings suggest that glycemic dysregulation and peripheral inflammation are independently associated with cognitive decline and structural MRI brain volumes. Additionally, persons with lower inflammation might be more vulnerable to cognitive decline through glycemic dysregulation. This emphasizes the importance of targeting both glucose control and inflammation to slow cognitive decline in individuals with glycemic dysregulation.